# hASH1在肺神经内分泌肿瘤中的表达及其临床意义

**DOI:** 10.3779/j.issn.1009-3419.2010.04.09

**Published:** 2010-04-20

**Authors:** 菲 李, 志永 钟, 锐 李, 鹤宇 黄, 丽君 王, 东晗 郑, 道荣 张

**Affiliations:** 1 110001 沈阳，中国医科大学基础医学院病理学教研室 Department of Pathology, College of Basic Medical Sciences, China Medical University, Shenyang 110001, China; 2 110001 沈阳，中国医科大学临床医学院 Clinical Medical College, China Medical University, Shenyang 110001, China

**Keywords:** Human achaete-scute homolog 1, 肺神经内分泌肿瘤, 肿瘤标记, 肺肿瘤, Human achaete-scute homolog 1, Lung neuroendocrine neoplasms, Tumor markers, Lung neoplasms

## Abstract

**背景与目的:**

*hASH1*（human achaete-scute homolog 1）基因在中枢神经系统、自主神经系统、肾上腺嗜铬细胞、甲状腺C细胞以及肺的神经内分泌细胞的早期发育中发挥重要作用。本研究旨在明确正常肺组织和各型肺肿瘤中*hASH1*基因的表达情况，分析其表达与肺神经内分泌标志物表达的相关性，初步探讨hASH1作为临床病理诊断肺神经内分泌肿瘤标志物的可能性。

**方法:**

采用免疫组化的方法检测正常肺组织、肺炎性假瘤、肺神经内分泌癌（典型类癌、非典型类癌、大细胞神经内分泌癌、小细胞癌）和肺非神经内分泌癌（鳞癌、腺癌、大细胞癌）中hASH1和神经内分泌标志物（Chromogranin A、Synaptophysin、CD56）的表达情况。采用Western blot和RT-PCR的方法检测肺鳞癌、腺癌和小细胞癌组织中hASH1的表达情况。

**结果:**

hASH1在正常肺组织、肺炎性假瘤、鳞癌、腺癌和大细胞癌中不表达；hASH1在典型类癌中的表达阳性率为12.5%（2/16），在非典型类癌中的表达阳性率为75%（15/20），差别有统计学意义（*P* < 0.01）；在大细胞神经内分泌癌中的表达阳性率为60%（6/10），在小细胞癌中的表达阳性率为77.5%（31/40），差别无统计学意义（*P* > 0.05）；hASH1的表达与Chromogranin A、Synaptophysin、CD56存在相关性（*P* < 0.05）。

**结论:**

hASH1对肺神经内分泌肿瘤具有高度特异性和敏感性，可能成为临床病理诊断肺神经内分泌肿瘤的标志物。

## Abstract (en)

**Background and objective:**

Human achaete-scute homolog 1 (*hASH1*) gene plays a critical role in development of the central nervous system, automatic nervous system, adrenal medullary chromaffin cells, thyroid C cells and pulmonary neuroendocrine cells. The aim of this study is to determine *hASH1* gene expression in the normal lung tissue and various types of lung tumors, to analyze whether its expression correlated with pulmonary neuroendocrine markers, and to explore the possibility of hASH1 as clinical pathological markers in the neuroendocrine tumors compared with previous neuroendocrine tumor markers.

**Methods:**

hASH1, Chromogranin A, Synaptophysin and CD56 expression were examined in lung tumor specimens (lung inflammatory pseudotumor, squamous cell carcinoma, adenocarcinomas, large cell carcinoma, typical carcinoids, atypical carcinoids, large cell neuroendocrine carcinomas and small cell lung carcinoma and corresponding normal lung specimens) using immunohistochemistry (S-P method). Western blot and reverse transcription polymerase chain reaction (RT-PCR) assay were applied to detect the expressions of hASH1 protein and mRNA in lung cancer tissues.

**Results:**

hASH1 expression was positive in 2/16 (12.5%) typical carcinoids, 15/20 (75%) atypical carcinoids, 6/10 (60%) large cell neuroendocrine carcinomas and 31/40 (77.5%) small cell lung carcinoma, respectively, but not in any normal lung tissue (0/10), lung inflammatory pseudotumor (0/49), squamous cell carcinoma (0/30), adenocarcinomas (0/30) or large cell carcinoma (0/20). There was a significant difference in hASH1 expression between typical carcinoids and atypical carcinoids (*P* < 0.01), but not in large cell neuroendocrine carcinomas and small cell lung carcinoma (*P* > 0.05). hASH1 expression highly closely correlated with Chromogranin A, Synaptophysin and CD56 expression (*P* < 0.05).

**Conclusion:**

hASH1 is a new kind of highly specific markers of pulmonary neuroendocrine tumours, and may be applied to clinical pathology diagnosis of the pulmonary neuroendocrine tumors.

*hASH1*（human achaete-scute homolog 1）基因是一个bHLH（basic helix-loop-helix）转录因子，首先从甲状腺髓样癌的cDNA文库中克隆出来，位于12q22-q23^[1, 2]^。在脊椎动物的发育过程中，*ASH1*基因通常只暂时性地表达于胚胎肺神经内分泌细胞的早期分化阶段，在终末分化标记物出现后其表达趋于沉默^[3]^。已有研究表明在具有神经内分泌分化的前列腺癌^[4]^、甲状腺髓样癌^[5]^、胃肠道神经内分泌癌^[6]^中有*hASH1*基因的表达。目前临床常用的非激素类神经内分泌肿瘤标志物有嗜铬粒蛋白A（Chromogranin A, CgA）、突触素（Synaptophysin, Syn）和CD56等。本实验通过免疫组织化学方法、Western blot和RT-PCR方法检测肺肿瘤和正常肺组织中*hASH1*基因的表达情况，探讨其作为更为特异和敏感的临床病理诊断肺神经内分泌肿瘤的标志物的可能性。

## 1 材料与方法

### 1.1 材料

收集166例肺癌组织蜡块（鳞癌30例，腺癌30例，大细胞癌20例，典型类癌16例，非典型类癌20例，大细胞神经内分泌癌10例，小细胞癌40例），其中35例取自辽宁省肿瘤医院，其余取自中国医科大学附属一院，49例肺炎性假瘤及10例癌旁正常肺组织蜡块取自中国医科大学附属一院。其中23例肺癌蜡块有新鲜组织标本，9例鳞癌、9例腺癌和5例小细胞癌新鲜组织均取自中国医科大学附属第一临床医院2008年5月-2009年5月手术切除标本，术中切除后立即置于-70 ℃冰箱保存，用于提取组织蛋白和RNA。

### 1.2 主要试剂和来源

浓缩型兔抗人ASH1多克隆抗体（ab38557）购自Abcam公司，即用型鼠抗人Chromogranin A单克隆抗体（MAB-0202）、鼠抗人Synaptophysin单克隆抗体（MAB-0078）、鼠抗人CD56单克隆抗体（RAB-0256）及即用型SP超敏免疫组织化学试剂盒（KIT-9710）和DAB酶底物显色试剂盒（DAB-0031）均购自福州迈新生物技术公司。ECL超敏发光液P1010和RIPA裂解液C1053购自普利莱基因技术公司。浓缩型辣根酶标记山羊抗兔（ZB-2301）、山羊抗鼠（ZB-2305）抗体均购自北京中杉金桥生物技术有限公司。RNA抽提试剂盒Trizol购自Invitrogen公司，RT-PCR反应试剂盒购自北京全式金生物技术公司，引物序列合成由南京金思特有限公司完成。

### 1.3 方法

#### 1.3.1 免疫组织化学检测

蜡块标本制成4 μm切片，经脱蜡、脱苯、水化后，采用链菌素抗生物素蛋白-过氧化物酶免疫组化法（SP法）检测，具体步骤参见试剂盒说明书进行，所有标本均检测hASH1（1:100）蛋白，所有肺神经内分泌癌标本均检测CgA、Syn和CD56的表达情况。每次染色均设立阳性对照，以PBS代替一抗做阴性对照。

#### 1.3.2 Western blot检测

从组织内提取细胞全蛋白, 取50 mg左右的组织块，加入500 μL裂解液中，冰浴下匀浆后，低温高速离心（4 ℃、12 000 g/min、20 min），收集上清。用考马斯亮兰法，以牛血清蛋白作为标准进行蛋白定量，计算各样品蛋白浓度。取总蛋白量80 μg上样，进行SDS-PAGE电泳分离。电泳条件：稳定电压80 V、30 min，待浓缩胶将蛋白质浓缩成一条直线后调至120 V、2 h，待溴芬兰进入凝胶底部后取出凝胶，根据预染Maker（Fermentas公司）标记切取所需相应分子量蛋白条带，将蛋白转印到PVDF膜上（40 V恒压，4 ℃湿转100 min），5%脱脂奶粉封闭2 h，加兔抗人ASH1抗体（1:500），4 ℃孵育过夜，TTBS（20 mmol/L Tris-HCL, 500 mmol/L NaCl, 0.05% Tween-20）漂洗3次后，加山羊抗兔（1:5 000）二抗温育120 min，TTBS漂洗3次后行ECL显色，X线胶片曝光成像，以β-actin为内参照，最后经自动电泳凝胶成像分析仪采集图像。

#### 1.3.3 RT-PCR检测

利用Trizol提取组织中总的RNA，具体操作步骤参见说明书进行，总RNA纯度和浓度使用紫外分光光度计（Nano Photometer, Germany）测定，经测定所用样品的*A*_260_/*A*_280_比值都在1.8-2.0之间。反转录过程按说明书操作。hASH1引物：上游序列5’-TCCCCCAACTACTCCAACGAC-3’，下游序列是5’-CCCTCCCAACGCCACTG-3’，产物大小为233 bp；内参β-actin引物：上游5’-TGGAATCCTGTGGCATCCATGA AAC-3’，下游5’-TAAAACGCAGCTCGATAACAGTCCG-3’，产物大小为250 bp。循环条件为：94 ℃预变性5 min，94 ℃变性30 s，55 ℃退火30 s，72 ℃延伸30 s，30个循环后于72 ℃延伸5 min，最后经琼脂糖凝胶电泳后保存图像。

### 1.4 结果判定

#### 1.4.1 免疫组织化学结果判定

hASH1在细胞核呈现棕黄色细小颗粒者为阳性染色，CgA、Syn在细胞浆呈现棕黄色细小颗粒者为阳性染色，CD56在细胞膜和细胞浆呈现棕黄色细小颗粒者为阳性染色。每张切片在400倍镜下观察10个视野，每个视野计数100个肿瘤细胞。阳性细胞数≤10%为1分，11%-50%为2分，50%-75%为3分，≥75%为4分；再按染色强度评分：无色为0，淡色为1分，棕黄色为2分，棕褐色为3分；最后按照乘积分数分为4个等级：“-”（0-2），“+”（3-5），“++”（6-8），“+++”（9-12）。染色结果判定：分数≥3定为阳性， < 3为阴性。

#### 1.4.2 Western blot结果判定

hASH1的条带在25 kDa处，用图像分析软件测定各样品灰度值，并取其与β-actin的比值作为相对表达量进行比较分析。

#### 1.4.3 RT-PCR结果判定

hASH1在233 bp处出现高亮条带，用图像分析软件测定各样品光密度值，并取其与β-actin的比值作为相对表达量进行比较分析。

### 1.5 统计学分析

应用SPSS 17.0统计软件进行数据处理，对hASH1在各种肺组织中表达的差异采用χ^2^检验（*n* > 40）或*Fisher*确切概率法（*n* < 40）分析，采用*Spearman*等级相关分析hASH1与CgA、Syn、CD56表达的相关性，*P* < 0.05为差异有统计学意义。

## 2 结果

### 2.1 免疫组织化学结果

#### 2.1.1 hASH1在正常肺组织、肺炎性假瘤和非小细胞肺癌中的表达情况

在10例正常肺组织、49例肺炎型假瘤、30例鳞癌、30例腺癌和20例大细胞癌中均无hASH1的表达。

#### 2.1.2 hASH1在类癌中的表达情况

在16例典型类癌中有2例阳性表达，阳性率为12.5%，在20例非典型类癌中有15例阳性表达，阳性率为75%（图 1），hASH1在典型类癌和非典型类癌中的表达差异有统计学意义（*P* < 0.01）。

**1:**
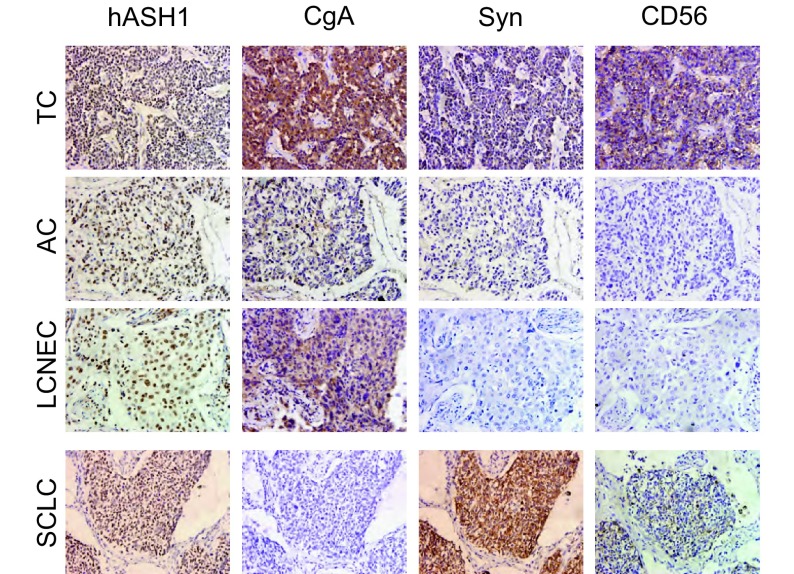
hASH1、CgA、Syn和CD56分别在TC、AC、LCNEC和SCLC中的表达情况（SP, ×400） Expression of hASH1, CgA, Syn and CD56 in TC, AC, LCNEC and SCLC respectively (SP, ×400)

#### 2.1.3 hASH1在大细胞神经内分泌癌和小细胞癌中的表达情况

在10例大细胞神经内分泌癌中有6例阳性表达，阳性率为60%，在40例小细胞癌中31例阳性表达，阳性率为77.5%（图 1），hASH1在大细胞神经内分泌癌和小细胞癌中的表达差异无统计学意义（*P*=0.42），hASH1在典型类癌和小细胞癌中的表达差异有统计学意义（*P* < 0.001）。

#### 2.1.4 神经内分泌标志物在肺神经内分泌癌中的表达及与hASH1的关系

在36例类癌中，27例（75.0%）CgA阳性，26例（72.2%）Syn阳性，29例（80.6%）CD56阳性。在50例大细胞神经内分泌癌和小细胞癌中，24例（48.0%）CgA阳性，38例（76.0%）Syn阳性，42例（84.0%）CD56阳性（图 1）。统计分析显示hASH1与CgA、Syn、CD56的表达均存在相关性（*P* < 0.05）（表 1）。

**1:** 在肺神经内分泌癌中hASH1与CgA、Syn、CD56表达的关系 Correlation of expression between hASH1and CgA, Syn, CD56 in pulmonary neuroendocrine carcinoma

		hASHl		*r*	*P*
		+	-		
CgA	+	38	l3	0.293	0.006
	-	l6	l9		
Syn	+	45	l9	0.265	0.014
	-	9	l3		
CD56	+	48	23	0.217	0.045
	-	6	9		

### 2.2 Western blot检测hASH1在肺鳞癌、腺癌和小细胞癌中蛋白的表达

在5例小细胞癌组织中均有*hASH1*基因的高表达，在9例鳞癌和9例腺癌组织中均无*hASH1*基因的表达（图 2）。

**2:**
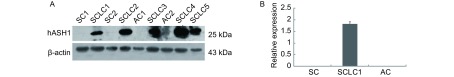
Western blot检测hASH1蛋白在肺癌组织中的表达 Expression of hASH1 protein in different lung cancer tissues by Western blot

### 2.3 RT-PCR检测hASH1在肺鳞癌、腺癌和小细胞癌中mRNA的表达

在5例小细胞癌组织中均有*hASH1*基因的表达，在9例鳞癌和9例腺癌组织中均物*hASH1*基因的表达（图 3）。

**3:**
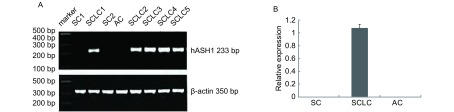
RT-PCR检测hASH1 mRNA在肺癌组织中的表达 Expression of hASH1 mRNA in different lung cancer tissues by RT-PCRA: RT-PCR results of hASH1 mRNA expression in squamous cell carcinoma, adenocarcinomas and small cell lung carcimoma, β-actin served as an internal control; B: The histogram of relative expression rate of hASH1 mRNA compared to β-actin. There is no expression of hASH1 mRNA in squamous cell carcinoma and adenocarcinomas, but in five small cell lung carcimoma the expression level is high.

## 3 讨论

*hASH1*基因编码含有238个氨基酸的前体蛋白，该蛋白与MASH1（mouse achaete-scute homolog 1, MASH1）蛋白有95%的同源性，主要通过结合靶基因启动子的E-box（核心序列为5′-CANNTG-3′）而启动基因的转录，该过程还需要与其它bHLH蛋白形成二聚体才能与DNA有效的结合^[1]^。hASH1在中枢神经系统、自主神经系统、肾上腺嗜铬细胞、甲状腺C细胞、肺神经内分泌细胞等多种组织细胞的早期发育中发挥重要作用^[1, 7, 8]^。

在鼠胚胎发育中敲除*ASH1*基因，会影响到神经内分泌分化，导致肺神经内分泌细胞的缺失，而肺神经内分泌细胞又与肺小细胞癌的发生有关^[9]^。相反，在转基因鼠中过表达ASH1能够促进气道上皮细胞的增生，ASH1与SV40（simian virus 40, SV40）大T抗原共同转染能够促进具有神经内分泌特征的肺肿瘤的形成^[10]^。最近，Osada等^[11]^研究发现，在有ASH1表达的肺癌细胞系中以RNAi的方式抑制ASH1的表达能够引起肿瘤细胞在G_2_-M期细胞周期停滞和细胞凋亡，其分子机制可能与半胱天冬酶-9和7的激活有关。人体活组织实时定量RT-PCR检测发现hASH1在各种神经内分泌肿瘤包括肺神经内分泌肿瘤、甲状腺髓样癌（medullary thyroid cancer, MTC）以及胎儿肺的神经内分泌细胞中都有表达，而在无神经内分泌细胞的肿瘤和正常组织中却未发现hASH1的表达^[12]^。这些均表明*hASH1*基因与具有神经内分泌分化特征的肿瘤密切相关。

由于典型类癌、非典型类癌和大细胞神经内分泌癌的发病率较低，我们未收集到这几种肺癌的新鲜标本，肺小细胞癌在临床上多采用非手术治疗，本实验也仅收集到5例新鲜标本，Western blot和RT-PCR的结果均显示在这5例小细胞癌中均有*hASH1*基因的高表达，而在9例鳞癌和9例腺癌中均无*hASH1*基因的表达。免疫组化结果也显示*hASH1*基因的表达仅限于具有神经内分泌特征的肺癌中，在正常肺组织、肺炎性假瘤、鳞癌、腺癌及大细胞癌中均未检测到*hASH1*基因的表达，这说明*hASH1*基因对肺神经内分泌肿瘤有高度特异性，可用于临床病理诊断。而且在除典型类癌外的其它肺神经内分泌癌中，*hASH1*基因的表达阳性率均较高，提示*hASH1*基因对诊断肺神经内分泌癌有较高的敏感性。另一方面，*hASH1*基因在典型类癌中表达较低（12.5%），而在非典型类癌和小细胞癌中的表达阳性率均很高（75%和77.5%），差异具有统计学意义。典型类癌具有较明显和成熟的内分泌表型，分化较高，一般无坏死，分裂像罕见，属于低度恶性，其生物学行为近似良性肿瘤，预后较好，5年生存率达90%以上；非典型类癌分化较典型类癌差，癌巢中有坏死，核分裂像多见，属于中度恶性，预后也较典型类癌差；小细胞癌是肺癌中分化最低、恶性程度最高的一种类型，生长迅速，转移早，5年生存率仅1%-2%，这些均提示*hASH1*基因的表达与肿瘤的分化程度密切相关，分化程度越低*hASH1*基因的表达阳性率越高，但*hASH1*基因能否作为判断患者预后的指标有待进一步的研究。

总之，*hASH1*基因对肺神经内分泌肿瘤具有高度特异性和敏感性，有可能成为肺神经分泌肿瘤尤其是肺小细胞癌的临床病理诊断标志物，其与CgA、Syn、CD56联合检测，有望将肺神经内分泌肿瘤的临床病理诊断提高到一个新的水平。

## References

[1] Ball DW, Azzoli CG, Baylin SB (1993). Identification of a human achaete-scute homolog highly expressed in neuroendocrine tumors. Proc Natl Acad Sci USA.

[2] Renault B, Lieman J, Ward D (1995). Localization of the human achaete-scute homolog gene (ASCL1) distal to phenylalanine hydroxylase (PAH) and proximal to tumor rejection antigen (TRa1) on chromosome 12q22-q23. Genomics.

[3] Ball DW (2004). Achaete-scute homolog-1 and development Notch in lung neuroendocrine and cancer. Cancer Lett.

[4] Rapa I, Ceppi P, Bollito E (2008). Human ASH1 expression in prostate cancer with neuroendocrine differentiation. Mod Pathol.

[5] Chen H, Kunnimalaiyaan M, Van Gompel JJ (2005). Medullary thyroid cancer: the functions of raf-1 and human achaete-scute homologue-1. Thyroid.

[6] Shida T, Furuya M, Nikaido T (2005). Aberrant expression of human achaetescute homologue gene 1 in the gastrointestinal neuroendocrine carcinomas. Clin Cancer Res.

[7] Guillemot F, Lo LC, Johnson JE (1993). Mammalian achaete-scute homolog 1 is required for the early development of olfactory and autonomic neurons. Cell.

[8] Huber K, Bruhl B, Guillemot F (2002). Development of chromaffin cells depends on MASH1 function. Development.

[9] Borges M, Linnoila RI, vande Velde HJ (1997). An achaete-scute homologue essential for neuroendocrine differentiation in the lung. Nature.

[10] Linnoila RI, Zhao B, DeMayo (2000). Constitutive achaete-scute homologue-1 promotes airway dysplasia and lung neuroendocrine tumors in transgenic mice. Cancer Res.

[11] Osada H, Tatematsu Y, Yatabe Y (2005). *ASH1* gene is a specific therapeutic target for lung cancers with neuroendocrine features. Cancer Res.

[12] Westerman BA, Neijenhuis S, Poutsma A (2002). Quantitative reverse transcription polymerase chain reaction measurement of HASH1 (ASCL1), a marker for small cell lung carcinomas with neuroendocrine features. Clin Cancer Res.

